# Racial and Ethnic Diversity Among Obstetrics and Gynecology, Surgical, and Nonsurgical Residents in the US From 2014 to 2019

**DOI:** 10.1001/jamanetworkopen.2021.9219

**Published:** 2021-05-19

**Authors:** Claudia L. López, Machelle D. Wilson, Melody Y. Hou, Melissa J. Chen

**Affiliations:** 1 Department of Obstetrics and Gynecology, University of California, Davis; 2 Division of Biostatistics, Department of Public Health Sciences, University of California, Davis

## Abstract

This cross-sectional study examines racial and ethnic diversity among obstetrics and gynecology (OBGYN), surgical, and nonsurgical residents in the US.

## Introduction

Health inequities are prevalent throughout US society. Within obstetrics and gynecology (OBGYN), Native American or Alaskan Native and non-Hispanic Black women are 3- to 4-fold more likely to have a pregnancy-related death compared with non-Hispanic White women.^1^ While addressing these disparities requires multiple strategies, one approach is to increase diversity among health care practitioners, as patient-physician racial/ethnic concordance is associated with increased patient satisfaction and higher levels of trust.^2^ Previous research, such as a 2020 study by Nieblas-Bedolla et al,^3^ indicates that the OBGYN workforce includes more underrepresented physicians compared with other specialties. By examining current and recent trainees, we can assess recruitment efforts and estimate the future racial and ethnic diversity of the physician workforce. In this cross-sectional study, we examine the contemporary composition and trends in race and ethnicity among OBGYN, surgical, and nonsurgical residents.

## Methods

The University of California, Davis, institutional review board determined that this cross-sectional study was not human participants research; therefore, it was exempt from review and informed consent. We followed the Strengthening the Reporting of Observational Studies in Epidemiology (STROBE) reporting guideline.

In this cross-sectional study, we abstracted deidentified publicly available data on the race and ethnicity of OBGYN, surgical, and nonsurgical residents from the *JAMA* Medical Education reports from 2014 to 2019, when *multiracial* first appeared as a racial category. We utilized the American College of Surgeons definition of a surgical specialty.^4^

We analyzed categorical data using χ^2^ tests and, for each race and ethnicity, used logistic regression to estimate the dependent variable as the change in odds of a resident identifying as a given race or ethnicity, across year and specialty. Given the small number of residents, we combined Native Hawaiian or Pacific Islander with Native American or Alaskan Native into a single Native category for analysis. We analyzed Hispanic ethnicity separately from race. We used SAS statistical software version 9.4 (SAS Institute) for statistical analysis. *P* values were 2-sided, and statistical significance was set at *P* < .05. Data were analyzed from 2014 to 2019.

## Results

A total of 520 116 US medical residents from 2014 to 2019 were included in this study. For each year, OBGYN, surgical, and nonsurgical residents most commonly identified as White (eg, 2014-2015: 58 098 residents [59.3%] ), followed by Asian (eg, 2014-2015: 26 010 residents [26.6%]) (Table). Native American or Alaskan Native and Native Hawaiian or Pacific Islander residents were the least represented in all residency categories (eg, 2014-2015: 325 residents [0.3%]). The racial and ethnic composition of residents varied among OBGYN, surgical and nonsurgical specialties each year, with higher proportions of OBGYN residents who identified as Black (eg, 2014-2015: 514 residents [10.2%]; *P* < .001) or Hispanic (eg, 2014-2015: 481 residents [9.6%]; *P* < .001) compared with surgical (eg, 2014-2015: 872 Black residents [4.7%]; *P* < .001; 1299 Hispanic residents [7.0%]; *P* < .001) and nonsurgical specialties (eg, 2014-2015: 4341 Black residents [5.8%]; *P* < .001; 5675 Hispanic residents [7.6%]; *P* < .001).

**Table.** Race and Ethnicity of OBGYN, Surgical, and Nonsurgical Resident Physicians per Academic Year, 2014 Through 2019

Specialty	Total, No.	Race/ethnicity, No. (%)							
		Hispanic ethnicity	White	Asian	Black	Native American or Alaskan Native	Native Hawaiian or Pacific Islander	Multiracial	Other or unknown
2014-2015									
OBGYN	5018	481 (9.6)	3400 (67.8)	727 (14.5)	514 (10.2)	9 (0.2)	9 (0.2)	177 (3.5)	182 (3.6)
Surgical^a^	18 521	1299 (7.0)	12 528 (67.6)	3652 (19.7)	872 (4.7)	41 (0.2)	23 (0.1)	544 (2.9)	861 (4.7)
Nonsurgical^b^	74 387	5675 (7.6)	42 170 (56.7)	21 631 (29.1)	4341 (5.8)	140 (0.2)	103 (0.1)	1896 (2.6)	4106 (5.6)
2015-2016									
OBGYN	5061	506 (10.0)	3442 (68.0)	724 (14.3)	495 (9.8)	9 (0.2)	4 (0.1)	163 (3.2)	224 (4.4)
Surgical^a^	19 067	1330 (7.0)	13 040 (68.4)	3795 (19.9)	863 (4.5)	41 (0.2)	16 (0.1)	489 (2.6)	823 (4.3)
Nonsurgical^b^	75 386	5756 (7.6)	42 772 (56.7)	21 698 (28.8)	4413 (5.9)	110 (0.1)	64 (0.1)	1907 (2.5)	4422 (5.9)
2016-2017									
OBGYN	5143	506 (9.8)	3430 (66.7)	739 (14.4)	468 (9.1)	9 (0.2)	6 (0.2)	176 (3.4)	315 (6.1)
Surgical^a^	19 476	1361 (7.0)	13 079 (67.2)	3870 (19.9)	863 (4.4)	34 (0.2)	18 (0.1)	575 (3.0)	1037 (5.3)
Nonsurgical^b^	77 850	5989 (7.7)	43 178 (55.5)	21 729 (27.9)	4455 (5.7)	90 (0.1)	97 (0.1)	2283 (2.9)	6018 (7.7)
2017-2018									
OBGYN	5346	542 (10.1)	3437 (64.3)	797 (14.9)	461 (8.6)	10 (0.2)	3 (0.1)	173 (3.2)	465 (8.7)
Surgical^a^	20 241	1444 (7.1)	13 351 (66.0)	4017 (19.9)	862 (4.3)	35 (0.2)	15 (0.1)	617 (3.1)	1344 (6.6)
Nonsurgical^b^	82 194	6562 (8.0)	44 462 (54.1)	22 608 (27.5)	4782 (5.8)	113 (0.1)	86 (0.1)	2454 (3.0)	7689 (9.4)
2018-2019									
OBGYN	5453	553 (10.1)	3532 (64.8)	833 (15.3)	431 (7.9)	8 (0.1)	8 (0.1)	195 (3.6)	446 (8.2)
Surgical^a^	21 098	1578 (7.5)	13 912 (65.9)	4101 (19.4)	890 (4.2)	28 (0.1)	24 (0.1)	689 (3.3)	1454 (6.9)
Nonsurgical^b^	85 875	7130 (8.3)	46 409 (54.0)	23 599 (27.5)	4953 (5.8)	120 (0.1)	120 (0.1)	2785 (3.2)	7889 (9.2)

Abbreviation: OBGYN, obstetrics and gynecology.

^a^ Surgical specialties include colon and rectal surgery, neurological surgery, ophthalmology, orthopedic surgery, otolaryngology, plastic surgery, plastic surgery-integrated, surgery-general, vascular surgery-integrated, thoracic surgery, thoracic surgery-integrated, and urology.

^b^ Nonsurgical specialties include allergy and immunology, anesthesiology, dermatology, emergency medicine, family medicine, internal medicine, medical genetics, neurology, nuclear medicine, osteopathic neuromusculoskeletal medicine, pathology-anatomy and clinic, pediatrics, physical medicine and rehabilitation, preventative medicine, psychiatry, radiation oncology, radiology-diagnostic, interventional radiology-integrated, transitional year, and combined specialties.

Among OBGYN residents, we noted a decrease in White (odds ratio [OR], 0.96; 95% CI, 0.94-0.98) and Black residents (OR, 0.93; 95% CI, 0.90-0.96) and an increase in those categorized as other or unknown race/ethnicity (OR, 1.26; 95% CI, 1.22-1.31) across the 5-year period (Figure). In surgical specialties, there were decreases in White (OR, 0.97; 95% CI, 0.97-0.98) and Black residents (OR, 0.97; 95% CI, 0.95-0.99) and increases in multiracial (OR, 1.04; 95% CI, 1.02-1.07) and other or unknown residents (OR, 1.14; 95% CI, 1.12-1.16). Lastly, among nonsurgical residents, there was a decrease in White (OR, 0.97; 95% CI, 0.96-0.97) and Asian residents (OR, 0.98; 95% CI, 0.97-0.98), while there was an increase in multiracial (OR, 1.07; 95% CI, 1.06-1.08), other or unknown (OR, 1.17; 95% CI, 1.16-1.18), and Hispanic residents (OR, 1.02; 95% CI, 1.02-1.03).

**Figure.**
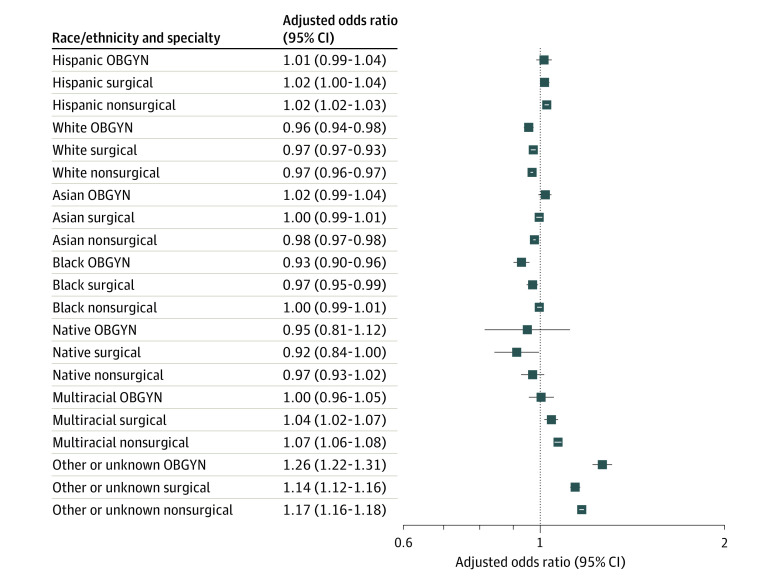
Adjusted Odds Ratios of Race/Ethnicity of Obstetrics and Gynecology (OBGYN), Surgical, and Nonsurgical Residents From 2014 to 2019 Native indicates Native American, Alaska Native, Native Hawaiian, or Pacific Islander.

## Discussion

The findings of this cross-sectional study suggest that while OBGYN residencies had higher proportions of Black and Hispanic residents compared with surgical and nonsurgical specialties, proportions of Black OBGYN residents decreased, while the proportion of Hispanic residents remained unchanged. Furthermore, our findings indicate that the diversity of OBGYN resident physicians still lagged behind the changing US demographic characteristics: non-Hispanic Black people represent 13% of the population, and Hispanic people represent 18% of the population, and both groups are projected to continue increasing in the coming years.^5^ As a diverse workforce is more likely to provide care for underserved populations and foster higher levels of trust, efforts to recruit and retain underrepresented individuals from racial/ethnic minority groups, such as Black, Hispanic, Native American or Alaskan Native, Native Hawaiian or Pacific Islander individuals, within OBGYN are critical as we strive to close gaps in health disparities.^2,6^

This study has some limitations, including that the database was comprised of self-reported data. Additionally, the other or unknown race/ethnicity category could indicate missing data or that residents did not identify with the listed racial/ethnic categories, thereby contributing to the downward trend in other racial categories.
